# Proteotoxic stress and the ubiquitin proteasome system

**DOI:** 10.1016/j.semcdb.2023.08.002

**Published:** 2023-09-19

**Authors:** Rachel Kandel, Jasmine Jung, Sonya Neal

**Affiliations:** a School of Biological Sciences, Department of Cell and Developmental Biology, University of California San Diego, La Jolla, CA 92093, United States; b Howard Hughes Medical Institute, Chevy Chase, MD, USA

**Keywords:** Ubiquitin, Proteasome, Ubiquitin proteasome system, Ubiquitin stress, Proteasome stress, Proteotoxic stress, Deubiquitinase, Protein misfolding, Aggregates, Neurodegeneration, Unfolded protein response, Usp14, Nrf1

## Abstract

The ubiquitin proteasome system maintains protein homeostasis by regulating the breakdown of misfolded proteins, thereby preventing misfolded protein aggregates. The efficient elimination is vital for preventing damage to the cell by misfolded proteins, known as proteotoxic stress. Proteotoxic stress can lead to the collapse of protein homeostasis and can alter the function of the ubiquitin proteasome system. Conversely, impairment of the ubiquitin proteasome system can also cause proteotoxic stress and disrupt protein homeostasis. This review examines two impacts of proteotoxic stress, 1) disruptions to ubiquitin homeostasis (ubiquitin stress) and 2) disruptions to proteasome homeostasis (proteasome stress). Here, we provide a mechanistic description of the relationship between proteotoxic stress and the ubiquitin proteasome system. This relationship is illustrated by findings from several protein misfolding diseases, mainly neurodegenerative diseases, as well as from basic biology discoveries from yeast to mammals. In addition, we explore the importance of the ubiquitin proteasome system in endoplasmic reticulum quality control, and how proteotoxic stress at this organelle is alleviated. Finally, we highlight how cells utilize the ubiquitin proteasome system to adapt to proteotoxic stress and how the ubiquitin proteasome system can be genetically and pharmacologically manipulated to maintain protein homeostasis.

## 1. Introduction

Proper protein function is crucial for maintaining normal cellular processes and health. For those functions to be carried out, proteins must be folded into their proper, native confirmations. Many challenges and disruptions, such as gene mutations, errors in protein translation, oxidative damage, and toxic environmental conditions, can occur during the process of protein folding [1–6]. These disruptions result in protein misfolding or non-native protein conformations. Because protein misfolding is a common occurrence in cells, cells have sophisticated mechanisms for recognizing and removing misfolded proteins. However, when these mechanisms fail, misfolded proteins accumulate, leading to proteotoxicity or cellular damage [7].

The abundance of specific proteins is a tightly controlled process in eukaryotic cells, with protein quality control (PQC) pathways employed to maintain protein homeostasis (proteostasis). The ubiquitin proteasome system (UPS) is the primary route by which proteins are degraded in eukaryotic cells [8]. The UPS is widely used to maintain cellular homeostasis by removing misfolded or damaged proteins, as well as controlling the levels of specific regulatory proteins [9–11 ]. The UPS is also utilized to remove unassembled subunits of larger protein complexes and to regulate the activity of cellular pathways by degrading proteins in the pathway, thus modulating pathway output. [12,13]. In this pathway, a small polypeptide, ubiquitin (Ub), is post-translationally conjugated to a target protein. Ub marks the protein for disposal by a large protein complex, the proteasome, which recognizes and proteolytically degrades target proteins (Table 1).

While proteotoxic stress can impact cells through a variety of mechanisms, a common mechanism is by causing dysfunction to the UPS. In turn, UPS impairment, either by pharmacological means, genetic alterations, or non-proteotoxic stressors, can also disrupt proteostasis. A properly functioning UPS is also critical in preventing and adapting to proteotoxic stress. If the UPS fails to prevent proteotoxic stress, cells are equipped with pathways that activate transcriptional programs which aim to restore proteostasis, including the unfolded protein response as well as the proteasome stress response in yeast and the proteasome recovery pathway in mammals. This review will explore proteotoxic stress as a cause of UPS dysfunction, primarily focusing on ubiquitin and proteasome stress, and the ability of the UPS to ameliorate proteotoxicity, with a focus on cell culture, mice, and yeast. The final section of this review will also focus specifically on quality control at the endoplasmic reticulum and the impacts of proteotoxic stress from this organelle. The review will also merge basic biology discoveries on PQC with our understanding of diseases where the UPS is affected.

### 1.1. Misfolded protein structures

Protein misfolding refers to a single protein that is not properly folded. Many times, misfolded proteins form larger structures of multiple interacting misfolded proteins, known as protein aggregates. Protein aggregation is often driven by exposed hydrophobic regions of proteins and high concentrations of aggregation-prone proteins can increase the likelihood of protein aggregation [14–16]. While hydrophobic stretches in proteins can increase the likelihood that a protein aggregates, there are no strict sequence requirements for protein aggregation [17,18]. Protein aggregation encompasses a wide variety of non-native multi--protein interactions and is examined in more detail in the review by Mogk, A., et al. [19].

Collectively, deleterious cellular effects caused by aggregated and misfolded proteins are referred to as proteotoxic stress. While some aggregates can be toxic and are implicated in the pathogenesis of many diseases, other aggregates are tolerated by cells or even serve adaptive purposes, such as transient aggregates that form under heat stress which protect the proteome [20,21]. Two mechanisms of toxicity by protein aggregates are 1) sequestration and subsequent inactivation of proteins by large aggregates and 2) inhibition of proteins by direct binding of small aggregates [22,23]. It has been appreciated for over two decades that misfolded protein aggregation can impair the UPS [24].

Protein aggregates can form various structures, such as soluble oligomers, amyloid fibrils, and amorphous aggregates (Fig. 2). While amyloid fibrils are a general histological indicator of neurodegenerative disease, existing evidence points toward soluble oligomer aggregates as a common driver of cytotoxicity [24,25]. Amorphous aggregates, such as inclusion bodies (IBs), are also commonly found in neurons in patients with neurodegenerative diseases, and are an adaptation by cells to sequester toxic oligomers, known as cytoprotective protein aggregation [26]. Ub is commonly found in IBs, but in most cases, IBs do not appear to be the cause of UPS dysfunction. UPS dysfunction is triggered before the formation of IBs, indicating IBs can be an adaptive response to proteasome impairment triggered by smaller, more cytotoxic aggregates [27]. Insoluble protein aggregates, like IBs, are generally resistant to proteasomal degradation and are instead disposed of by autophagy, another PQC pathway [28].

### 1.2. Protein misfolding diseases and the UPS

Diseases that are driven by misfolded and aggregated proteins are known as protein misfolding diseases[2]. Some of these diseases, like cystic fibrosis, are loss of function diseases driven by specific protein mutations that cause misfolding and target the protein for degradation[29,30]. Other protein misfolding diseases, most notably many neurodegenerative diseases, result in amyloids of specific proteins, but are not usually caused by mutations in the aggregated protein. This latter category of protein misfolding diseases has a variety of genetic risk factors associated with each disease[31,32]. Protein aggregation is a common theme among neurodegenerative diseases and is a well-studied context in which proteotoxic stress and its impact on the UPS has been researched **(**Table 1**).** As such, much of the research reviewed here will be in relation to neurodegeneration (highlighted in Section 2.1.1 Proteasome Stress in Neurodegeneration).

### 1.3. The ubiquitin proteasome system machinery

#### 1.3.1. Ubiquitin

Ubiquitination is a post-translational modification commonly used to target proteins for degradation by the proteasome [33]. Ub is a small (76 amino acid), tightly folded polypeptide that can be covalently attached to a target protein through an isopeptide bond, most frequently on lysine residues, and less frequently to cysteine, serine, and threonine residues [34]. Ubiquitination as a post-translational modification can be used for targeting proteins for proteolytic degradation, as well as other signaling purposes, such as triggering a DNA damage repair response, autophagy, and endocytosis [35]. This review will focus primarily on ubiquitination as a signal for degradation.

Proteins can either be monoubiquitinated or tagged with a chain of Ubs, called polyubiquitination. Monoubiquitination is most commonly used as signaling modification on proteins, most notably in regulating the DNA damage response [36–38]. Generally, proteins targeted for degradation become polyubiquitinated, meaning subsequent Ubs are conjugated to the first Ub through the lysine residues of Ub. The polyubiquitin (polyUb) chains that target proteins to degradation are usually chains of at least four Ub proteins [39]. Ub contains 7 lysine residues (K6, K11, K27, K29, K33, K48, and K63), and the C-terminal of one Ub can be conjugated to any of the lysine residues of another Ub. As an example of the nomenclature used to describe Ub linkages, a Ub chain where each Ub is attached through the K63 residue would be a K63 linkage and a chain where some Ubs are conjugated to K11 and some to K63 would be called a mixed linkage chain. Different lengths of Ub chains and different linkage types cause different cellular effects, known as the Ub code [5]. For example, the K48 linkage is most commonly associated with protein degradation [40], although, in yeast, cytoplasmic UPS targets must have both K11 and K48 linkages to be degraded [41].

In addition to Ub, eukaryotic cells also utilize ubiquitin-like proteins (UBLs) [42]. While there are many UBL families expressed in mammalian cells, this review will touch on the role of the UBL SUMO (small--ubiquitin-related modifier) in SUMOylation and NEDD8 in neddylation (Section 2.2.2 Friends of Ubiquitin: Ubiquitin-like proteins in Stress).

#### 1.3.2. The ubiquitin symphony: E1s, E2s, E3s, and deubiquitinases in concerts

**Fig. 1.**
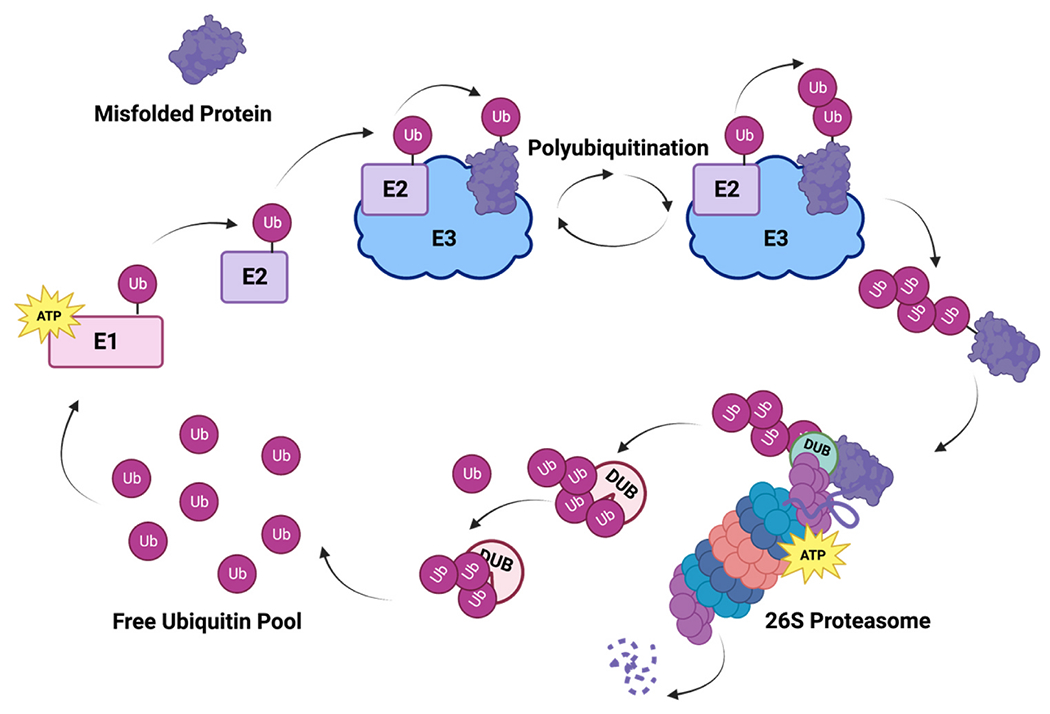
The Ubiquitin Proteasome System. Ub is initially conjugated to an E1 ubiquitin activating enzyme, in an ATP-dependent reaction, and is then transferred to an E2 Ub conjugation enzyme. E3 ligases bring an Ub-E2 complex in proximity with a target protein, to which Ub gets transferred. Additional ubiquitins are conjugated through the same processes to create a polyUb chain. This chain targets proteins to the 26 S proteasome, where deubiquitinases (DUBs) remove Ub before ATP-dependent proteasomal degradation. Other DUBs specifically break down Ub chains to replenish the cellular pool of free ubiquitin, which is then accessible for E1 enzymes. Created with BioRender.com.

**Fig. 2.**
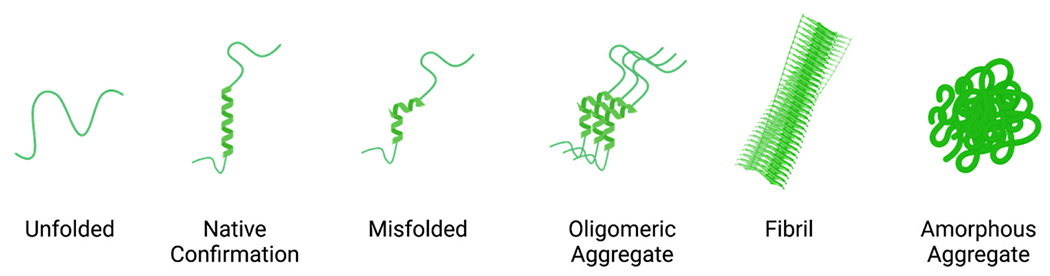
Misfolded Protein Structures Graphical depiction of an unfolded protein, native conformation protein, and several misfolded protein structures that are referenced throughout this review. Created with BioRender.com.

Essential players in the UPS are the enzymes in the conjugation cascade that enables Ub to be attached to target proteins, the enzymes responsible for removing Ub from proteins, and the enzymes that break down Ub chains (Fig 1). For Ub to be conjugated to a target protein, it must first go through a cascade of E1 ubiquitin-activating enzymes, E2 ubiquitin-conjugating enzymes, and E3 ubiquitin ligases. First, Ub is conjugated to an E1 ubiquitin-activating enzyme by a thioester bond on a cysteine residue in an ATP-dependent manner [43,44]. Next, Ub is transferred to a cysteine residue on an E2 ubiquitin-conjugating enzyme [45,46]. Finally, an E3 ubiquitin ligase binds the ubiquitinated E2 enzyme and the target protein to promote ubiquitination of the target protein [47–49]. For some E3 ligases, Ub is first transferred to the E3 before being transferred to the target protein [50]. Once an initial Ub is added to a target protein, the cycle can be repeated to conjugate additional Ubs to the first and create a polyUb chain.

In total, the human genome contains over 800 genes encoding UPS components, including hundreds of E1, E2, and E3 enzymes [51]. The human genome encodes two E1 enzymes, about 40 E2 enzymes, and about 600 E3 ligases, giving layers of specificity to which target proteins are ubiquitinated by UPS machinery. UBLs also use their own set of E1, E2, and E3 enzymes, with some instances in which they are conjugated by ubiquitin-specific enzymes [52].

Just as E1s, E2s, and E3s are specialized for attaching ubiquitin to target proteins, deubiquitinases (DUBs) are proteases that specifically cleave the isopeptide bonds formed by Ub. Different DUBs have specificity for certain types of Ub linkages and have specific roles in Ub cleavage, such as removing entire polyUb chains or breaking down polyUb chains into monomeric Ubs [53,54]. DUBs play important roles in recycling Ub, regulating Ub chain lengths, and modulating the rate of degradation of UPS targets[55–59].

#### 1.3.3. The proteasome

The proteasome is a large protein complex found in both the cytoplasm and nucleus that degrades proteins [60]. The 26 S proteasome, which is the proteasome configuration that degrades ubiquitinated proteins, consists of two major components, the 20 S core particle (proteolytic core) and the 19 S regulatory particle (proteasome cap), with a proteasome cap on either side of the core [61–64].

The 20 S core forms a narrow cylinder (maximum of 53Å) and can only be entered by unfolded proteins [65]. It is composed of 4 stacked rings, each made out of 7 distinct subunits [61]. Of these 4 rings, the 2 inner rings are made of seven β subunits, three of which have protease activity, while the two outer rings are made of ɑ subunits, which lack a proteolytic function [61,66]. The 20 S core of the proteasome has trypsin-like, chymotrypsin-like, and caspase-like proteolytic activity, with each activity provided by a different proteolytic β subunit [67,68]. These various proteolytic functions allow the proteasome to progressively cleave most proteins into small peptide fragments in an ATP-dependent manner, with the ATPase activity provided by the 19 S proteasome [69]. To regulate protein degradation, the ɑ subunits composing the outer rings of the core form a gate channel with their N-terminals [61,70]. The gating mechanism ensures that only unfolded proteins trigger conformational changes that allow substrates to enter the core.

The cap of the proteasome serves to recognize ubiquitinated substrates, remove Ub, unfold target proteins, and translocate unfolded proteins into the proteolytic core [71–73]. The 19 S regulatory particle is composed of 19 subunits and two rings [74]. The base (the portion that interacts with the 20 S core particle) is made of 6 distinct ATPase subunits and 3 non-ATPase subunits [75]. Besides providing the energy for translocating unfolded proteins into the core, the ATPase units of the regulatory particle base, Rpt1–6 in yeast (Psmc2, Psmc1, Psmc4, Psmc6, Psmc3, and Psmc5 in humans, respectively), are also responsible for changing the conformation of the gate in the 20 S core to an open conformation [76,77]. Two of the non-ATPase subunits of the base, Rpn1 and Rpn2 in yeast (Psmd2 and Psmd1 in humans, respectively), serve as binding sites for Ub while the third non-ATPase subunit, Rpn13 (Adrm1 in humans), is a Ub receptor [75,78]. The top ring, or lid, is composed of 9 subunits, primarily structural proteins and one DUB, Rpn11 in yeast (Psmd14 in humans) [79]. The final subunit of the regulatory particle, Rpn10 in yeast (Psmd4 in humans), essentially connects the lid and the base [80,81].

In addition to the subunits mentioned above, the regulatory particle also associates with additional DUBs, Usp14 (Ubp6 in yeast) and Uch37 in humans [82–85]. The role of proteasome-associated DUBs is critical for protein degradation by the proteasome, as the regulatory particle only allows ubiquitinated proteins to be translocated to the core particle, but Ub is not necessary after initially being recognized by Ub receptors in the regulatory particle [86]. In fact, Ub remaining on proteasome targets during degradation can slow protein turnover, due to delayed unfolding of target proteins [58]. DUBs allow Ub to be removed from proteins, so that the Ub does not get degraded with the rest of the protein, thus recycling Ub for continued use in the cell [59,87,88].

## 2. Proteotoxic stress impacting the ubiquitin proteasome system

**Fig. 3.**
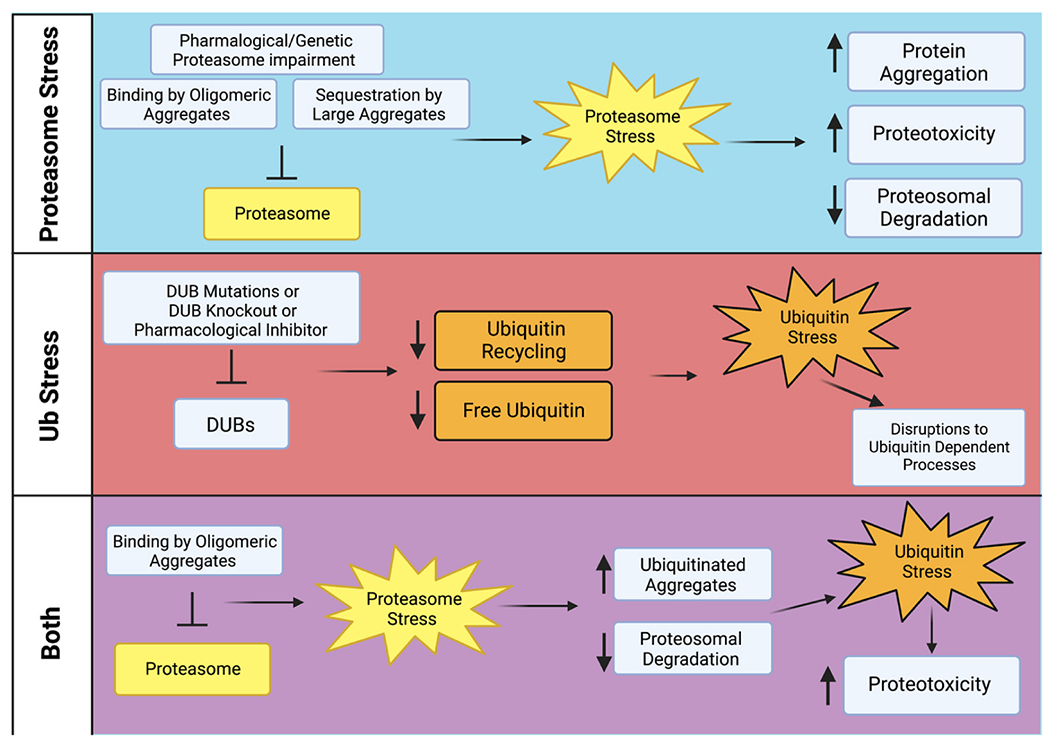
Causes and Impacts of Proteasome and Ubiquitin Stress. Top panel: Overview of causes of proteasome stress and the cellular impacts of proteasome stress. Middle panel: Overview of cellular impacts of impairment to DUBs and consequential ubiquitin stress. Bottom panel: Overview of how proteotoxic stress can lead to proteasome stress, which in turn can lead to ubiquitin stress. Created with BioRender.com.

Proteotoxic stress can impact the UPS directly, through the interaction of protein aggregates with UPS machinery, or as a downstream effect through non-direct mechanisms. In this section, we will examine the impacts of misfolded and aggregated proteins on bot**h** proteasome stress and ubiquitin stress (Fig. 3) [59]. For this review, we are defining proteasome stress as any reduction in proteasome activity that results in inefficient clearance of proteasome substrates. We are defining ubiquitin stress as a state where the amount of unconjugated Ub in the cell is altered to a level that normal cellular processes involving Ub are impacted. The terms proteasome stress and ubiquitin stress were coined by Daniel Finley’s lab in an influential 2007 *Cell* paper [49].

### 2.1. Proteasome stress

#### 2.1.1. Proteasome stress in neurodegeneration

Alzheimer’s disease (AD) is a neurodegenerative disorder in which patients present with extracellular protein deposits (aggregates) in the brain, followed by neuronal cell death. Amyloid β (Aβ) deposits are one of the hallmarks of AD and one of the common Aβ isoforms, Aβ42, is neurotoxic when in a soluble, oligomeric aggregated state (Table 1) [89]. Aβ is proteolytically derived from Amyloid Precursor Protein (APP), through consecutive processing by the proteases β-secretase 1 followed by γ-secretase [90]. The normal physiological function of APP is not completely understood, but existing evidence shows a role for APP and secreted Aβ in normal neuronal processes, such as neuronal cell migration during development, synaptic function, and transcriptional regulation [91–93]. Aβ42 causes proteasome inhibition in vitro [94], and proteasome function is reduced in the brains of AD patients and in AD mouse models [95,96]. Notably, the decrease in proteasome activity in humans and mice is not accompanied by a reduction in proteasome expression [95,96]. Aβ42 applied extracellularly to rat neuroblastoma cells (B103), cells that do *not* endogenously produce Aβ42, results in proteasome impairment, as measured through a proteasome-targeted GFP reporter [97]. Even when applied extracellularly, a portion of Aβ42 enters the cytoplasm of the cell, possibly indicating a direct proteasome impairment mechanism. In support of Aβ42 driving neurotoxicity by proteasome inhibition, genetically or pharmacologically increasing proteasome activity results in protection against cell death in mouse, fly, and cell culture Aβ models of AD [98].

In addition to Aβ aggregates, tau aggregates are another hallmark of AD that are neurotoxic and have been demonstrated to cause proteasome impairment (Table 1). Outside of the context of AD, tau is a protein involved in microtubule assembly [99]. Hyperphosphorylation of tau disrupts its normal function and promotes tau fibril formation [100–102]. Tau is normally a UPS substrate in healthy cells, and accumulation of tau results in a buildup of ubiquitinated tau, proteasome impairment, and proteasome interaction with tau, indicating that tau inhibits proteasomes directly [103]. Pharmacological proteasome activation, using the FDA-approved drug cilostazol, reduces the accumulation of ubiquitinated tau [104]. Explored further in section **2.2.1** Causes and Impacts of Ubiquitin Stress, Aβ can also reduce free Ub, demonstrating the interconnectedness of proteasome and ubiquitin stress, and the ability of a single aggregated protein to have polytropic impacts on the UPS.

Parkinson’s disease (PD) is a neurodegenerative disease where dopaminergic neurons of the substantia nigra decay and the disease is characterized by aggregated protein formations in the substantia nigra, known as Lewy Bodies. Lewy bodies contain ɑ-synuclein and Ub (Table 1) [105]. Soluble ɑ-synuclein localizes to synapses, but its normal function in the cell is unclear and has been proposed to be involved in many processes, see the review by Bendor et. al. for a comprehensive review of proposed functions of α-synuclein [106]. While most PD cases are sporadic with no clear genetic cause, many mutations in the UPS have been implicated in familial PD from genetic studies in humans, including in the deubiquitinase UCH-L1 and the E3 ligase, Parkin [107,108].

Impacting the proteasome, either with genetically or pharmacologically, can recapitulate PD in animal models. PSMC1 is a gene that encodes Rpt2, one of the ATPase subunits of the 19 S regulatory particle, and is critical in opening the gate of the core particle for substrate entry [74,76]. In PSMC1 forebrain-specific and substantial nigra-specific conditional knockout (cKO) mouse models, neurons depleted for PSMC1 primarily contained 20 S proteasomes, which do not degrade ubiquitinated proteins [109]. These mice developed neurodegeneration, as measured by a reduction in cortical thickness and neuronal cell death [109]. The substantia nigra PSMC1 cKO mice showed massive neuronal loss and an accumulation of Ub in surviving neurons. The neurons depleted for PSMC1 also formed Lewy body-like inclusions [109]. This study indicates that 26 S proteasome dysfunction can lead to a PD-like neurodegenerative disease, pointing to proteasome dysfunction as a potential precursor of neurodegeneration. In addition to a genetic model for PD caused by proteasome dysfunction, chemical models of PD have been established by injecting the proteasome inhibitor lactacystin into the forebrain of mice, rats, and minipigs [110–112]. It was demonstrated more recently in these PSMC1 cKO mice that autophagy components become downregulated at the gene level upon long-term proteasome dysfunction, leading to a buildup of ubiquitinated mitochondrial proteins, ubiquitinated by Parkin [113]. This study points to defective mitophagy and/or mitochondrial protein degradation as a potential driver of neurodegeneration in this proteasome-defective PD model (PSMC1 cKO), which is in line with autophagy defects observed in other PD models [114].

#### 2.1.2. Mechanisms of proteasome impairment by aggregates

For the past ~30 years, it has been understood that many disease-associated protein aggregates can bind proteasomes in vitro and impair proteasome function [94,115,116]. However, the mechanism of impairment occurring and whether it varies from protein to protein was not well understood. The Smith Lab at West Virginia University has established that oligomeric aggregates of a range of protein misfolding disease-associated proteins all have a similar mechanism of proteasome impairment (Table 1) [23]. The proteins tested were oligomers of α-synuclein (associated with PD), Aβ (associated with AD), and Huntingtin 53Q (Htt53Q: a mutant protein that causes Huntington’s disease (HD). This research found oligomeric aggregates of intermediate size bind directly to the proteasome in vitro and allosterically inhibit proteasomal degradation by holding the proteasome gate in a closed conformation[23]. In contrast, the large aggregates and small oligomers did not impact the ability of the proteasome to degrade a fluorescent proteasome substrate. Prior to this work, it was already demonstrated that small oligomers of Aβ tend to be more cytotoxic than higher molecular weight aggregates, but the mechanism was unclear [117]. Pairing these findings with the knowledge that proteasome activity is reduced with age in many cell types [118,119], the Smith Lab created the first transgenic animal model with an open-gate proteasome [120]. Using *C. elegans* with a genetic perturbation to express a partially open-gate proteasome, the lab determined these organisms had a longer lifespan and better ability to cope with oxidative and proteotoxic stress, although this came at the cost of a massive reduction in fertility compared with wildtype (WT) worms. Increased lifespan has also been demonstrated in *C. elegans* with overexpression of the 19 S proteasome subunit Rpn-6 and these worms have increased proteasome activity and resistance to proteotoxic stress [121]. Similarly, protein aggregates form in aged yeast (*S. cerevisiae)* and can be reduced in a genetic background where proteasome components are upregulated [122].

While the above work implicates oligomeric aggregates as direct inhibitors of proteasomes, some aggregated protein structures may impair proteasomes in vivo. A recent study demonstrated that purified α-synuclein can form different aggregated structures under different conditions [123]. When these aggregates were injected into mouse brains and then examined by immunohistochemistry, only α-synuclein fibrils that were arranged in a structure where the C-terminus was exposed became ubiquitinated, phosphorylated, and sequestered proteasomes in intracellular aggregates [123]. All those characteristics are features of α-synuclein fibrils in PD, indicating that the C-terminal exposed fibril conformation could be a driver of toxicity in vivo.

Amyotrophic lateral sclerosis (ALS) is a neurodegenerative disease characterized by the death of motor neurons and cytoplasmic aggregates containing ubiquitinated TAR DNA-binding protein 43 (TDP-43), an RNA processing protein (Table 1). Several causative mutations for ALS have been reported in proteins that promote proteasomal degradation, including the ATPase p97/VCP, which is required for the degradation of select UPS substrates [124–126]. The most common causative mutation for familial ALS is a hexanucleotide expansion in the *C9orf72* gene. One result of this mutation is the production of aggregated poly-glycine alanine (polyGA) C9orf72 peptides, which are toxic to neurons (Table 1) [127]. Cryo-electron tomography of poly-GA peptides in neurons reveals the peptides form a twisted ribbon-like structure, in which proteasomes become stalled while attempting to degrade the aggregated peptides and become sequestered as a result of this stalling [22]. In contrast to the mechanism identified for oligomeric aggregates, this is an example of a mechanism by which larger protein aggregates could directly impair proteasomes [22,23]. Similarly, glycine-rich C-terminal fragments of TDP-43, known as TDP-25 itself can form aggregated inclusions, which cryo-electron tomography has revealed also physically sequester stalled proteasomes [128].

#### 2.1.3. Cellular adaptations to proteasome stress

One of the major mediators of cellular adaptation to proteasome stress is the transcription factor Rpn4 in yeast and Nrf1 in mammals [129,130]. Rpn4 and Nrf1 are both transcription factors that primarily upregulate proteasome component genes. The transcriptional program orchestrated by Rpn4 in yeast is called the proteasome stress response (PSR) [131], and Nrf1 activates a similar program in mammalian cells called the proteasome recovery pathway [130]. Our group (the Neal lab at University of California, San Diego) and others have identified Rpn4 as a key mediator in adapting cells to misfolded membrane protein accumulation at the ER [132,133], as well as being crucial in adaptation to stressors that induce protein aggregation and certain forms of proteasome inhibition stress [131]. Rpn4 is a cytoplasmic protein that is normally rapidly degraded by the proteasome, both in a ubiquitin-dependent and -independent manner [134,135]. Upon proteasome stress, impairment of the proteasome naturally slows the degradation rate of Rpn4, causing an increase in Rpn4’s stability and abundance [129]. The increased pool of Rpn4 then accumulates in the nucleus, where it upregulates many proteasome components at the mRNA level, eventually leading to increased proteasome capacity [129,136]. In contrast to the activation of Rpn4, Nrf1 is instead an ER-localized membrane protein that is proteolytically processed into an active transcription factor in response to proteasome impairment [130,137–139].

As an example of the impact of Rpn4 in adaptation to stress, stabilizing Rpn4 in yeast by knockout (KO) of Ubr2, the E3 ligase that ubiquitinates Rpn4, results in increased replicative lifespan in yeast, and this effect is specific to the increase in proteasome capacity in these mutants [140]. While in this example increasing proteasome capacity in aging cells is beneficial, there are examples in human diseases where the proteasome recovery pathway should be attenuated, such as in cancer. Proteasome inhibitors, such as bortezomib and carfilzomib, are frequently prescribed for the treatment of multiple myeloma and induce apoptosis of multiple myeloma cells [141,142]. To determine if multiple myeloma cells depend on the proteasome recovery pathway, the Bianchi Lab at Brigham and Women’s Hospital used multiple human myeloma cells lacking Ddi2, the protease that activates Nrf1, and found that these cells were less viable and more sensitive to proteasome inhibition [143]. This provides a new strategy by which multiple myeloma cells that acquire proteasome inhibitor resistance can be targeted.

The PSMC3 gene encodes Rpt5, an ATPase subunit of the proteasome which also recognizes polyUb chains [74]. There is a rare heterozygous monogenic human disease caused by a mutation in PSMC3 that results in impaired proteasome function [144,145]. Patients with this mutation are deaf and blind, with neurological deficits. In patient-derived fibroblasts, there is a build-up of ubiquitinated proteins and an increase in the amount of 26 S proteasome. Despite increased levels of proteasomes, there was no increase in proteolytic capacity, indicating some fraction of the proteasome is non-functional [144]. In the patient-derived cells, the reduction in proteosome activity triggers constitutive activation of Nrf1. As a result, the Nrf1 pathway becomes exhausted and cannot be further activated by proteotoxic stress, which can be deleterious to the cells and is a potential cause of this developmental disorder [144]. The role of Nrf1 in increasing UPS capacity in response to proteasome stress demonstrates the adaptability of cells to proteotoxic stress. Understanding the importance of this pathway for proper cellular function and cell survival could guide our understanding of rare diseases with defects in proteasome function.

#### 2.1.4. Causes and impacts of ubiquitin stress

Ub has wide-ranging roles in cellular functions and, as such, the amount of Ub must be well-regulated. Because of the process by which Ub is conjugated to proteins, the only Ub that is readily usable is monomeric, referred to as free Ub. The amount of free Ub in cells is known as the free Ub pool. When the free Ub pool is reduced to a level that disrupts ubiquitin-dependent processes, this state is ubiquitin stress [59].

When human cells in culture undergo heat shock, a form of stress that causes proteins to misfold, the cells accumulate ubiquitinated proteins and show a reduction in free Ub [146]. When cells begin to recover from heat shock, they continue clearing soluble ubiquitinated proteins and increase the expression of Ub. In contrast, aggregated proteins that become ubiquitinated during heat stress are not cleared, posing a potential threat to cells in the aftermath of cellular stress [146].

Because of the many roles of Ub, depletion of free Ub can have wide-ranging cellular consequences, even outside of proteostasis. Both a mutant protein that causes HD (HttQ91) and a mutated, aggregation-prone luciferase can form IBs containing Ub [147]. Because of the sequestration of Ub by these IBs, there is less monoubiquitination of histones, which is a signaling function of Ub that regulates the DNA damage response. As a result, the DNA damage response is impaired in the cells that have HttQ91 or luciferase IBs. This is an example of how ubiquitin stress, triggered by proteotoxic stress, can have downstream cellular effects outside of the proteostasis network.

In addition to the previously described characteristic of Aβ42 to impair proteasomes, Aβ42 applied extracellularly to primary neurons results in reduced free Ub. As a result, this causes a reduction in cell viability and an increase in the mRNA level of several inflammatory markers [148]. This indicates ubiquitin stress may be one of the drivers of cytotoxicity by Aβ42 aggregates, in addition to causing proteasome stress.

Ub is commonly found in protein aggregates and ubiquitinated aggregates are a hallmark of protein misfolding diseases [149]. Two types of ubiquitinated intracellular protein inclusions are the juxtanuclear quality control compartment (JUNQ) and insoluble protein deposit (IPOD) [150]. JUNQ inclusions are hubs of proteasomal degradation, whereas IPOD inclusions generally contain terminally aggregated ubiquitinated proteins [150]. The Yerbury group at Illawarra Health and Medical Research Institute has extensively studied the impact of aggregation-prone proteins associated with ALS on Ub dynamics [151,152]. The lab initially characterized the types of aggregates formed by mutants of three proteins implicated in ALS (Table 1). The proteins interrogated in this study were superoxide dismutase 1 (SOD1), an enzyme with antioxidant functions, TDP-43, an RNA-processing protein, and Fused in Sarcoma (FUS), an RNA-binding protein [153,154]. SOD1 forms JUNQ inclusions while TDP-43 and FUS can form JUNQ or IPOD-like inclusions, indicating the exact type of protein inclusion formed is not common to all cases of ALS [155]. The group’s research has determined, in NSC-34 cells (mouse motor neuron-like immortalized cells), that disease-associated mutants of SOD1, TDP-43, and FUS all form aggregates that include Ub conjugates with K48 and K63 linkages. The aggregated forms of mutant SOD1 and FUS result in reduced UPS activity and a reduction in free Ub, in a manner that does not occur with the WT form of each protein or because of the non-aggregated mutant protein. In contrast, aggregated WT and mutant TDP-43 both reduce UPS activity when overexpressed, but only aggregated mutant TDP-43 specifically results in decreased free Ub [155].

#### 2.1.5. Friends of ubiquitin: ubiquitin-like proteins in stress

While ubiquitination is the most common post-translational modification to target proteins for degradation, mammalian cells also encode a collection of ubiquitin-like proteins (UBLs) that can also target proteins for proteasomal degradation. One UBL, SUMO, plays a role in modulating stress granule dynamics [156]. Stress granules are cytoplasmic amorphous aggregates of RNA and protein that form under conditions of cellular stress, as an adaptive response to sequester cytotoxic oligomeric aggregates [157]. SUMOlyation can act as a primer for ubiquitination, which is carried out by the SUMO-targeted ubiquitin ligase (StUbL) pathway. Under stress conditions, RNA binding proteins, which are commonly included in stress granules, in the nucleus become SUMOlyated and are then recognized by the Ub E3 ligase Rnf4, a component of the StUbL pathway. These now ubiquitinated proteins targeted by the StUbL pathway are then degraded by the nuclear proteasome. If the StUbL pathway is impaired, stress granules will still form, but their disassembly is slowed [156]. Another recent study investigating the ubiquitome under various stress conditions found that ubiquitination of proteins in stress granules was required for stress granule disassembly, but only when the stress granules were induced by heat shock and not by arsenite [158]. In contrast to the work on StUbL on stress granules, this study demonstrated that ubiquitination was important because it allowed for recruitment of the ATPase p97/VCP to disassemble stress granules [158].

Another UBL, NEDD8, has been shown in human cell culture to serve an adaptive role under conditions of cellular stress, by promoting cytoprotective protein aggregation and sparing UPS function as a result [159] . Specifically, when cells are heat shocked, the ribosomal protein RPL7 becomes both NEDDylated and ubiquitinated, resulting in a poor proteasome substrate compared to ubiquitination alone. Thus, the mixed Ub/NEDD8 chains are less efficient than ubiquitination chains alone. This slowing of RPL7 degradation results in nuclear stress granules but prevents nuclear proteasome function from becoming overwhelmed [159].

#### 2.1.6. Deubiquitinases in mediating ubiquitin stress

DUBs are proteases that specifically cleave the isopeptide bonds formed by Ub, and act on specific linkage types or chain lengths, including releasing Ub chains from ubiquitinated proteins or breaking down Ub chains into Ub monomers [53,54]. Several DUBs regulate the pool of free Ub by recycling Ub on proteins degraded by the proteasome [160]. This includes one of the DUBs that interacts with the proteasome lid, Usp14 in mammals and Ubp6 in yeast [59,88,161].

Ubp6 is important for cell survival in circumstances where proteotoxicity disrupts free Ub. Prions are a type of misfolded proteins that are highly toxic and induce misfolding and aggregation of properly folded proteins [123,162]. Yeast Ubp6 KO cells expressing prions exhibit cellular stress, as measured by a reduction in growth compared to WT yeast [88]. This growth stress seems to be related to the inability of Ubp6 KO cells to efficiently recycle Ub, as increasing the amount of monomeric Ub in Upb6 KO cells with prions restores growth. Ubp6 is important for ubiquitin recycling, because this DUB releases Ub from UPS-targeted proteins before proteasomal degradation, rather than allowing Ub to be degraded along with the targeted protein [84]. Proteasomal degradation of Ub as a driver for Ub depletion in these Ubp6 KO cells is also supported by the observation that Ub was stabilized when the proteasome was inhibited in these cells [88]. The absence of Ubp6 also increases the sensitivity of cells to treatment with amino acid analogs that cause disruptions to proteostasis [88]. Our lab has also observed growth stress in Ubp6 KO yeast expressing a misfolded membrane protein, as well as in cells lacking other DUBs, indicating a broad role for DUBs in maintaining Ub homeostasis and in adapting yeast cells to proteotoxic stress [132].

A mouse with a loss of function mutation in the Ubp6 homolog, Usp14, displays neurodegeneration and ataxia. This mutation, an insertion in intron 5 of Usp14, causes a large reduction in the expression of Usp14 and the mice have reduced neurotransmitter release and low synaptic transmission [163]. Initially puzzling, the neurons of these mice do not have ubiquitin-containing protein aggregates [163]. This mutation causes polyUb chains to be degraded rather than recycled, which in turn lowers the amount of free Ub in neurons without causing direct impairment to proteasomes [161]. The decreased amount of free Ub is the primary reason for this neurological dysfunction, demonstrating that ubiquitin stress can be a driver of cellular dysfunction in the absence of protein aggregation [161]. In these mice, neurological dysfunction by ubiquitin stress can be relieved through the influx of free Ub and restored expression of Usp14. Restoring Usp14 and increasing expression of monomeric Ub in ataxic mice restores synaptic function, motor function, and muscle mass to WT levels [164–167]. The benefits of increasing free Ub in ataxic mice may have a narrow therapeutic window, as too much Ub results in additional neurological dysfunction, decreasing muscle mass, and impairing motor nerve function [168].

Injecting mice with a Usp14 inhibitor results in the inhibition of long-term memory, as measured through fear response behavioral tests [169]. In Usp14-deficient ataxic mice, presynaptic transmission and signaling from the hippocampus to the amygdala are impaired, although this effect appears to be caused by a protease-independent function of Usp14 [170,171]. This decrease in presynaptic transmission with a Usp14 genetic mutant potentially explains the long-term memory deficits in mice treated with a Usp14 inhibitor.

Usp14 itself acts as a regulator of proteasome activity, with overexpression of Usp14 inhibiting proteasome function [172]. The E3 ligase Trim11 can bind to Usp14, thereby preventing the interaction between Usp14 and the proteasome. This interaction increases proteasome activity, including the ability of the proteasome to degrade aggregated proteins. This increase in proteasome activity can be adaptive for cancer cells, as Trim11KO in a xenograft cancer mouse model reduces tumor growth significantly and overexpression of Trim11 in the same model has the inverse effect [172].

Two DUBs have been implicated in stress granule dynamics; Usp5 and Usp13 [173]. The Komada Lab at the Tokyo Institute of Technology used HeLa cells, fluorescence, and super-resolution microscopy to determine that K48- and K63-linked Ub chains co-localize in stress granules formed by heat stress. Stress granule dynamics are regulated by these Ub chains, and the chains are regulated by stress granule-associated Usp5 and Usp13. Usp5KO or Usp13KO results in faster assembly and slower disassembly of stress granules, meaning these DUBs antagonize the formation of stress granules. However, the role of polyUb chains in stress granule dynamics is disputed. Work from Eric Bennett’s lab at University of California, San Diego has demonstrated that inhibiting ubiquitination does not impact stress granule dynamics, and the majority of Ub in stress granules is actually monomeric Ub [174]. This demonstrates a circumstance where monomeric Ub is not necessarily reduced in cells, but rather free Ub is physically sequestered and could result in ubiquitin stress.

## 3. The endoplasmic reticulum and the ubiquitin proteasome system

### 3.1. Protein quality control at the ER and proteotoxic stress responses

**Fig. 4.**
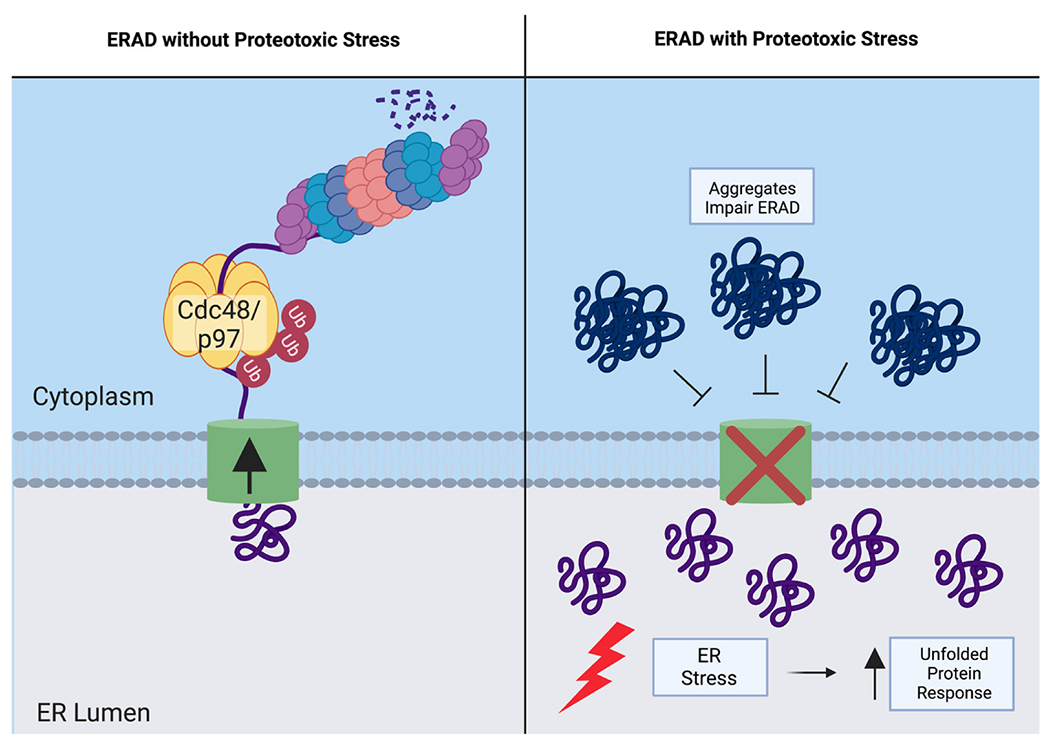
Endoplasmic Reticulum Associated Degradation and Proteotoxic Stress. The left panel of the figure depicts the role of ERAD under normal physiological conditions. Misfolded proteins are retrotranslocated from the ER lumen into the cytoplasm, in a ubiquitin-dependent process that requires a retro-translocon and the ATPase p97/VCP in mammalian cells and Cdc48 in yeast cells. The right panel depicts that proteotoxic stress from protein aggregates can lead to impairment of ERAD. This impairment increases proteotoxic stress specifically at the ER and leads to activation of the UPR, a pathway that aims to restore ER proteostasis. Created with BioRender.com.

The endoplasmic reticulum (ER) is a major site of protein synthesis and protein folding, specifically for secreted proteins and membrane proteins. The UPS pathway utilized specifically at the ER is called ER-associated degradation (ERAD) [9,175]. The ER is a well-studied site of PQC by the UPS and it is well-understood how proteotoxic stress is handled at the ER [176]. The canonical ER stress response to misfolded protein accumulation is the unfolded protein response (UPR). The UPR is the best-studied cellular adaptation pathway for proteotoxic stress. This section of the review will explore the interplay between the UPR and ERAD function (Fig. 4).

#### 3.1.1. Endoplasmic reticulum associated degradation and ubiquitin proteasome system impairment

Compared to cytoplasmic protein quality control by the UPS, there are additional challenges to UPS degradation of ER-localized proteins. The ER is responsible for folding secreted proteins destined for the plasma membrane and secretory pathway resident proteins [177]. When ER proteins are degraded by ERAD, they must be moved across the ER membrane for degradation by the cytoplasmic proteasome, a process termed retrotranslocation [178,179]. A universal feature of ERAD in both yeast and mammalian cells is the use of an ATPase, p97/VCP in mammals and Cdc48 in yeast, to provide the energy for retrotranslocation and to mechanically assist with protein unfolding before proteasomal degradation [180–182].

Several neurodegenerative diseases, most famously HD, are caused by abnormally long stretches of polyglutamine in specific proteins (known as polyQ expansions). When expressed in yeast and in neuronal-like PC12 cells (derived from rats), a polyQ expanded Huntingtin protein (103Q Htt exon 1) quickly impairs ERAD (6 h in yeast and 8 h in PC12 cells), prior to the onset of global UPS impairment. This impairment of ERAD by polyQ aggregates is toxic and driven by the sequestration of critical ERAD components (Table 1). For example, p97 and Cdc48 are sequestered by these aggregates in PC12 cells and yeast cells, respectively [183]. Overexpressing the sequestered ERAD components promotes cell survival [183]. Additional evidence that UPS is impaired in HD comes from the observation that K48 Ub linkages accumulate globally with the expression of a GFP-tagged polyQ Htt variant (HttQ150GFP) in a mouse neuroblastoma cell line [184]. However, in a HD mouse model, UPS function is not impaired globally in the brain [185]. While there are changes in protein ubiquitination in these mice, these changes are not the same as in mice injected with a proteasome inhibitor [186]. One proposed explanation for this discrepancy is that ubiquitination may be impacted in specific neuronal cell types that contribute to HD, rather than globally. Impairment of ERAD, particularly by polyQ expanded proteins, can be caused independently of impairment to the UPS as a whole and can be a driver of toxicity.

#### 3.1.2. Rhomboid proteins: the bridge between ERAD and proteotoxic stress

A major class of proteins that participate in ERAD is the rhomboid protein family. Rhomboid proteins are intramembrane proteins with two subclasses: rhomboid proteases and rhomboid pseudoproteases, with the pseudoproteases lacking a proteolytic active site. In both yeast and mammalian cells, ER-localized rhomboid proteins have a role in the retrotranslocation step of ERAD [179,187–189]. The mammalian rhomboid protease RHBDL4 can cleave aggregation-prone proteins and direct the fragments to degradation by the proteasome [190]. The Neal Lab has recently established the ability of rhomboid *pseudoproteases* to similarly act on aggregation-prone membrane proteins, with yeast rhomboid pseudoprotease Dfm1 preventing misfolded membrane protein aggregation [132]. When membrane proteins aggregate in the absence of Dfm1, it is toxic to the cells. This toxicity appears to be primarily driven by ubiquitin stress, as the free Ub pool is reduced by roughly half and the cells grow normally when Ub expression is increased [132]. Remarkably, in cells lacking Dfm1, expressing the mammalian homologs of Dfm1, Derlin-1 or Derlin-2, restores the ability of yeast cells to prevent membrane protein aggregation and prevents ubiquitin stress [132].

Several mammalian rhomboid proteins that participate in ERAD are also involved in stress alleviation. Derlin-1 is expressed in 66% of breast cancer tumors, but it is not expressed in healthy mammary cells [191]. This rhomboid protein is also upregulated by ER stress in normal cells and appears to play a protective role against apoptosis in breast cancer [191]. Protection against apoptosis by ERAD is generally accepted due to the ability of this degradation pathway to ameliorate proteotoxic threats to the cell and promote cellular health. Derlin-1 is also implicated in ALS through its interaction with mutant SOD1. This interaction causes an impairment to ERAD that results in ER stress and leads to motor neuron death [192]. Overexpression of Derlin-1 promotes clearance of SOD1, through both proteasomal degradation and autophagy, as well as reduces ER stress and apoptosis [193]. In cardiomyocytes, Derlin-3 is upregulated several-fold in response to ER stress, and its upregulation enhances ERAD and prevents apoptosis [194]. Additionally, ERAD appears to be critical in adapting cardiomyocytes to ER stress, as knockdown of the ER-localized E3 ligase Hrd1 increases apoptosis when ER stress is chemically induced in these cells [195]. The rhomboid protease RHBDL4 also plays a protective role against ER stress, as RHBDL4 KO mice are more sensitive to ER stress-inducing drugs than WT mice, and develop liver steatosis, a disease that can be induced by chronic ER stress [196].

#### 3.1.3. The unfolded protein response and the ubiquitin proteasome system

The UPR is a well-investigated response pathway to proteotoxic stress at the ER. The pathway can be activated by the accumulation of misfolded soluble ER proteins. The UPR in mammalian cells utilizes three ER membrane-localized sensors for activation; Ire1, PERK, and Atf6 [197–202]. All three sensors, through different mechanisms, result in downstream activation of gene networks that seek to restore proteostasis (for a thorough review of the UPR, please refer to Hetz, C., et al.) [203].

The UPR is a powerful pathway for adapting cells to stressors when activated transiently, however, constitutive activation can cause additional stress and cellular dysfunction, leading to apoptosis in mammalian cells [204]. Treatment of multiple myeloma cells with a proteasome inhibitor is an example of how apoptosis because of UPR activation can be leveraged for disease treatment. Treating these cells with a proteasome inhibitor causes an accumulation of misfolded ER proteins and leads to a maladaptive UPR response which triggers apoptosis [205].

Because ERAD specifically degrades misfolded proteins at the ER, it is closely intertwined with the UPR. In fact, the UPR causes upregulation of many ERAD components in mammalian cells, including Derlin-2, Derlin-3, and Hrd1, and allows for ERAD to more effectively degrade proteins [194,206–208]. Additionally, some mammalian ER E3 ligases can be stabilized by ER stress prior to the upregulation of ERAD components in order to quickly increase ERAD capacity. This is caused by autoubiquitination and degradation of the E3 ligase gp78, with degradation of other ER E3 ligases being slowed as a result of reduced ubiquitination from gp78 [209].

It has been appreciated for nearly two decades that ER stress can result in impairment of the UPS [210]. In yeast without Hac1, the transcription factor activated by Ire1, the rate of degradation of a misfolded protein at the ER is reduced [211]. However, the degradation of a misfolded cytosolic protein is not impacted, indicating unresolved ER stress impacts ERAD, but not the UPS as a whole[211].

Neuroinflammation is a potential driver of both aging and neurodegenerative diseases. Using lipopolysaccharide (LPS) injection in the hippocampus of rats as a model of neuroinflammation, the Ruano group at Universidad de Sevilla found that the Ire1 and Atf6 branch of the UPR are acutely activated by neuroinflammation, but attenuated by ~12 h post injection [212]. As a result of this attenuation, ERAD components become downregulated, resulting in defective ER quality control. It will be important in future studies to understand how PQC defects as a result of neuroinflammation affect cellular health and survival.

Aggregation of human islet amyloid polypeptide (hIAPP) from pancreatic β-cells is another illustrative example of how proteotoxicity, the UPR, and the UPS are connected in disease pathogenesis. hIAPP deposits occur extracellularly and are characteristic of Type 2 diabetes in. However, the deposition of hIAPP outside the cell triggers the UPR, both in a cell culture and mouse model of Type 2 diabetes [213,214]. In addition to UPR activation, cells also show UPS impairment, as evidenced by a buildup of ubiquitinated proteins, and this dysfunction appears to be due to prolonged ER stress in pancreatic β-cells [214]. Increasing proteasome activity increases cell viability in the presence of hIAPP [214]. Exogenous hIAPP expression in mice reduces the expression of UCH-L1, a DUB required for proteasomal degradation, at the protein and mRNA level. This downregulation of UCH-L1 promotes apoptosis due to UPS impairment [215]. It is unclear whether ER stress directly causes the downregulation of UCH-L1, or whether UCH-L1 downregulation is caused by another mechanism resulting in the promotion of ER stress through UPS dysfunction [215]. The proteasome itself is also able to degrade hIAPP, meaning that proteasome impairment could lead to an increase in hIAPP accumulation. However, through a negative feedback loop, proteasome impairment decreases the transcription and secretion of hIAPP in pancreatic β-cells [216].

## 4. Conclusions and perspectives

The ubiquitin proteasome system (UPS) is one of the major protein quality control pathways in eukaryotic cells and is critical for preventing and adapting to proteotoxic stress. Proteotoxic stress and the UPS are closely interconnected, as proteotoxic stress impairs the UPS and UPS dysfunction leads to proteotoxic stress (Fig. 3). Cells are finely tuned to prevent proteotoxic stress, but proteotoxic stress can easily overwhelm the UPS and lead to proteostasis failure.

Modulating the UPS to adapt cells to proteotoxic stress is a promising strategy for potential therapeutics. While proteasome inhibitors are currently approved for some cancers to induce apoptosis [141,142], increasing UPS capacity could also be harnessed to enhance cell survival in diseased cells, such as in neurodegenerative diseases. There are two potential strategies for increasing UPS capacity: 1) increasing proteasome activity and 2) increasing the pool of free ubiquitin [120,132,140,164]. In addition, cells use several pathways to adapt to proteotoxic stressors and modulate UPS capacity, the main pathways being the unfolded protein response, the proteasome recovery pathway (in mammals), and the proteasome stress response (in yeast) [130,131,176]. Modulation of these pathways will be a valuable strategy for treating a myriad of protein misfolding diseases.

Neurodegenerative diseases are examples of well-studied protein misfolding diseases in which the UPS is frequently impaired. While large amyloids or insoluble protein deposits are characteristics of several neurodegenerative diseases, smaller oligomeric aggregates are usually the drivers of cytotoxicity [23–26]. For several disease-causing proteins, oligomers have been shown to impair proteasome function and bind directly to proteasomes in in vitro *experiments* [23]. It will be an important line of inquiry to understand how UPS dynamics are affected during the early stages of neurodegenerative diseases in animal models.

## Figures and Tables

**Table 1:** Protein Misfolding Diseases and Associated Aggregates and Sources of Proteotoxicity. The table indicates the protein misfolding diseases, aggregated proteins associated with each disease that are discussed in this review paper, as well as evidence and causes of proteotoxic stress caused by the aggregates associated with each disease.

Term	Abbreviation
Alzheimer’s Disease	AD
Amyloid Precursor Protein	APP
Amyloid β	Aβ
Amyotrophic Lateral Sclerosis	ALS
Conditional Knockout	cKO
Deubiquitinase(s)	DUB(s)
Endoplasmic Reticulum	ER
ER-associated Degradation	ERAD
Fused in Sarcoma	FUS
Htt53Q	Huntingtin 53Q
Human Islet Amyloid Polypeptide	hIAPP
Huntingtin	Htt
Huntington’s Disease	HD
Inclusion Bodies	IBs
Insoluble Protein Deposit	IPOD
Juxtanuclear Quality Control Compartment	JUNQ
Knockout	KO
Lipopolysaccharide	LPS
Parkinson’s Disease	PD
Poly Glycine Alanine	polyGA
Polyglutamine	polyQ
Polyubiquitin	polyUb
Proteasome Stress Response	PSR
Protein Quality Control	PQC
Small-Ubiquitin-Related Modifier	SUMO
SUMO-targeted Ubiquitin Ligase	StUBL
Superoxide Dismutase 1	SOD1
TAR DNA-binding Protein 43	TDP-43
Ubiquitin	Ub
Ubiquitin Proteasome System	UPS
Ubiquitin-like protein(s)	UBL(s)
Unfolded Protein Response	UPR
Wildtype	WT
